# Follow‐Up After Receiving Abnormal Results From Self‐Sampled Colorectal and Cervical Cancer Screening Tests Among Underserved Patients

**DOI:** 10.1002/cam4.71283

**Published:** 2025-09-27

**Authors:** Jennifer L. Moss, Veronica E. Bernacchi, Juliette Entenman, Heather Costigan, Heather L. Stuckey, Mack T. Ruffin

**Affiliations:** ^1^ Penn State College of Medicine Hershey Pennsylvania USA; ^2^ Michigan State University East Lansing Michigan USA

**Keywords:** cancer screening, cervical cancer, colorectal cancer, self‐sampling, underserved patients

## Abstract

**Introduction:**

Increasing cancer screening through at‐home self‐sampling test modalities is a public health priority. Patients with abnormal screening results should receive diagnostic follow‐up care; optimizing this process is a challenge. We conducted a mixed methods study to examine the cancer screening process among underserved patients who received abnormal results on a self‐sampled cancer screening test.

**Methods:**

Participants were drawn from a parent study examining the impact of self‐sampled colorectal and cervical cancer screening tests among patients at federally qualified health centers in Pennsylvania. Those who had received abnormal results on their screening were completed a survey and semi‐structured interview about their experience (*n* = 5). We conducted mixed methods analysis to examine participants' (1) understanding and follow‐up care for abnormal results and (2) satisfaction with the self‐sampling cancer screening process.

**Results:**

Quantitatively, participants indicated very high satisfaction with each self‐sampled cancer screening, and 60% preferred a self‐sampled test for their next cancer screening. Qualitatively, participants differed in the extent to which they seemed to understand their screening results, but they were generally satisfied with the self‐sampling process. In mixed methods analysis, participants' baseline knowledge about cancer screening supported better understanding of abnormal screening results, and participants' preference for their next cancer screening was related to their experiences with self‐sampling.

**Conclusions:**

Among this sample of patients who received abnormal results on their self‐sampled colorectal or cervical cancer screening test, knowledge and understanding were not prerequisites for accessing follow‐up care. Satisfaction with the self‐sampling screening process was very high. These findings provide additional support for public health priorities to expand access to self‐sampling cancer screening tests.

Follow‐up care is necessary for patients who receive abnormal cancer screening results. However, follow‐up care after abnormal cancer screening is suboptimal [1, 2, 3], especially for patients in vulnerable communities, where barriers to care include provider shortages and low health insurance coverage [1, 2]. Failures in follow‐up care can increase the likelihood of morbidity and mortality [1, 3].

Certain cancer screening tests are available through at‐home self‐sampling modalities. For colorectal cancer, the US Preventive Services Task Force (USPSTF) guidelines include at‐home stool tests, such as the fecal immunochemical test (FIT) [4], but use [5] and follow‐up [2] on these tests are low. For cervical cancer, USPSTF guidelines do not currently include a self‐sampling option; however, self‐sampling for high‐risk human papillomavirus (hrHPV) testing is highly accurate [6] and acceptable [7]. Receipt of follow‐up care after abnormal hrHPV tests is suboptimal [6]. Because self‐sampling tests are collected outside of the clinical setting, healthcare providers and systems have concerns about how best to connect patients to appropriate follow‐up procedures to achieve diagnostic resolution of abnormal cancer screening results [8, 9].

We investigated experiences after receiving abnormal results on a self‐sampled cancer screening test (i.e., FIT or hrHPV test) among female patients from federally qualified health centers (FQHCs), drawn from a parent study focused on increasing cancer screening in vulnerable communities [10, 11]. These findings have implications for integrating self‐sampling cancer screening tests into routine clinical care.

## 1 Materials and Methods

### 1.1 Parent Study

Participants came from a trial examining self‐sampling tests for cancer screening [10, 11]. Participants were recruited from nine FQHCs in six rural and racially segregated counties in Pennsylvania. Participants were female, ages 50–65, with an intact cervix, and eligible but out of date for both cervical and colorectal cancer screenings [4, 12]. Participants completed self‐sampled FIT and hrHPV testing, and their results were communicated back to participants' FQHCs. For participants with abnormal screening results, a physician at the FQHC called them within 14 days to discuss their results and recommendations for follow‐up care.

### 1.2 Procedures for the Current Study

Participants in the current study were participants from the parent study who received abnormal results on their self‐sampled FIT and/or hrHPV tests. After physician notification of abnormal results, the study team confirmed that the participant had received their screening results and obtained verbal informed consent for the current study.

Data collection focused on the experiences of participants with abnormal screening results. Outcomes of interest included [1] understanding and following up on screening test results, and [2] satisfaction with the self‐sampling screening process. We assessed these outcomes via survey and semi‐structured interview, administered via phone or HIPAA‐compliant teleconferencing platform. Data collection took an average of 22.4 min to complete (7.6 min for the survey and 14.9 min for the interview). Survey responses were entered into REDCap [13], and interviews were audio‐recorded, de‐identified, and transcribed verbatim.

Overall, 48 participants enrolled in the parent study, of whom 24 received the self‐sampling cancer screening tools. Of these, 7 participants had abnormal results (FIT: *n* = 4; hrHPV: *n* = 3), five of whom completed the current study (reasons for non‐participation: unreachable: *n* = 1; uninterested: *n* = 1). Participants received $30 for completing the current study.

### 1.3 Measures

#### 1.3.1 Baseline Surveys From the Parent Study

Sociodemographic variables included race/ethnicity, annual household income, educational attainment, and marital status. Healthcare variables included whether participants had a preventive health check‐up in the last year and health insurance type.

We assessed cancer screening knowledge using four true‐or‐false questions from the National Cancer Institute's Health Information National Trends Survey [14]. Similar to previous studies, we summed the number of correct responses to create an index ranging from 0 to 4, with higher scores indicating greater knowledge. We assessed health literacy with the 4‐item Brief Health Literacy Screening Tool [15]. Using standard procedures, we summed the responses to create a score ranging from 4 to 20, with higher scores indicating greater health literacy.

#### 1.3.2 Surveys From the Current Study

We measured satisfaction with each self‐sampling cancer screening test (separately for FIT vs. hrHPV testing) with four items: if they (1) thought the test was easy to use, (2) liked that they could do the test themselves, (3) thought the test was private, and (4) thought the test was convenient [16]. We summed the number of “yes” responses to create an index ranging from 0 to 4, with higher scores indicating greater satisfaction. We also assessed preference for the next cancer screening test; response options for were self‐sampling test at home, test at the doctor's office, no preference, both tests, or other, which we collapsed into self‐sampling test at home vs. other.

#### 1.3.3 Semi‐Structured Interview From the Current Study

We developed a semi‐structured interview guide informed by existing literature on cancer screening. The interview guide consisted of open‐ended questions with suggested follow‐up probing questions to elicit rich, descriptive narratives about the self‐sampling cancer screening intervention delivery, content, and follow‐up (Table S1). All interviews were conducted via telephone and audio recorded with participant consent.

### 1.4 Analytic Methods

Analysis followed a concurrent triangulation design of quantitative, qualitative, and mixed methods analysis [17].

#### 1.4.1 Quantitative Analysis

We generated descriptive statistics for the sociodemographic variables of parent study participants in the self‐sampling arm (*n* = 24) and the current study participants (*n* = 5). We used Fisher's exact tests to assess differences in sociodemographic characteristics between samples. Then, we descriptively summarized current study participants' responses for quantitative measures.

#### 1.4.2 Qualitative Analysis

Descriptive qualitative analysis [18] was consistent with the framework method [19]. First, all audio‐recorded transcripts were transcribed, de‐identified, and cleaned. Next, the research team immersed themselves in the data by reading each transcript several times and wrote memos to note initially emerging patterns. The research team developed a deductive, a priori coding strategy based on the domains of intervention delivery, content, and follow‐up, which was applied to the dataset. The research team also applied a second, inductive coding strategy to the first two transcripts, then both inductive and deductive coding strategies were combined for the final coding schema. This final coding schema was iteratively updated as the team coded the final three transcripts, and then the final coding schema was applied to the entire dataset. The research team combined similar codes to develop categories, then assimilated like categories to develop descriptive themes by charting data into a framework matrix [19]. Qualitative rigor was upheld through regular peer‐debriefing meetings, an audit trail of analysis decisions, and contextualization of the results within the broader self‐sampling cancer screening literature [20, 21]. Besides peer‐debriefing, the analysts attained coding calibration with a final kappa of 0.76.

#### 1.4.3 Mixed Methods Analysis

We used the quantitative data to triangulate our qualitative findings. To do this, we combined findings from quantitative and qualitative data using joint displays [22]. The joint displays illustrate, separately for each participant, related concepts assessed during the surveys and the interviews. Finally, we sought to identify convergence or divergence between quantitative and qualitative findings [17].

Analyses were conducted using SAS version 9.4 and nVivo software. The Pennsylvania State University Human Research Protection Program approved this project.

## 2 Results

### 2.1 Quantitative Findings

Participants in the current study were similar to participants in the parent study (Table S2, all *p* > 0.05). Participants in the current study were predominantly non‐Hispanic white (80%), had < $50,000 annual household income (80%), and had greater than a high school degree (80%).

On average, participants scored 2.6 (standard error [SE] = 0.2) on the cancer screening knowledge index and 17.8 (SE = 0.7) for health literacy (Table 1). Satisfaction with the self‐sampling tests was uniformly high (mean = 4.0 for each screening). Most participants indicated a preference for taking a self‐sampling test at home the next time they needed screening (cervical cancer = 60%; colorectal cancer = 60%).

**TABLE 1:** Summary statistics for key quantitative variables related to understanding and satisfaction with self‐sampling for cancer screening among underserved patients.

	Mean	SE
**Baseline survey from the parent study**		
Cancer screening knowledge (range: 0–4)	2.6	0.2
Health literacy (range: 4–20)	17.8	0.7
**Survey from the current study**		
Satisfaction with self‐sampling cancer screening test		
Colorectal cancer (range: 0–4)	4.0	0.0
Cervical cancer (range: 0–4)	4.0	0.0
	***n***	**%**
**Preference for next cancer screening test**		
Colorectal cancer		
Self‐sampling test at home^a^	3	60%
Other	2	40%
Cervical cancer		
Self‐sampling test at home^a^	3	60%
Other	2	40%

Abbreviation: SE = standard error.

^a^ Preferences for ‘self‐sampling test at home’ reflect only participants who indicated that option only (i.e., excludes participants who selected ‘no preference’ or ‘both’ tests).

### 2.2 Qualitative Findings

We developed two themes about the self‐sampling cancer screening process.

#### 2.2.1 Theme 1: Understanding of Self‐Sampled Screening Results and Receipt of Appropriate Follow‐Up Care

Although participants were mixed in their understanding of self‐sampled cancer screening test results, all reported engaging with physicians to receive follow‐up care. Three participants used vague or general statements when discussing their FIT and/or hrHPV test results. In this example, Participant E (abnormal cervical cancer screening) indicated a superficial conversation about the results:

[The healthcare provider] just said the results were [abnormal]. And I took that at face value and didn't ask any further questions, which I probably should have. (Participant E; abnormal cervical cancer screening)

In contrast, two participants demonstrated a clear understanding of what abnormal screening test results meant. In this example, Participant C (abnormal colorectal cancer screening) summarized the results of both of her screening tests:

That [I] did not have the necessary markers for cervical cancer and had blood in my stool, but that could be from hemorrhoids. It could be, you know, lot of other things. (Participant C; abnormal colorectal cancer screening)

Understanding of cancer screening results was sometimes related to participants' family experiences and/or medical training. For example, one participant indicated that a family member's cancer experience provided her with some knowledge of the importance of cancer screening:

Well, they told me that that was less than normal progression of this, and I have an uncle who died of colon cancer. And I know that when you catch that early that most of the time you can live through it. (Participant B; abnormal colorectal cancer screening)

Similarly, another participant shared that her family and occupational experiences influenced her understanding of screening results:

I worked in the medical field. I was an emergency medical technician, and my own life experience between the health issues with my children, you know, I have a basic understanding. (Participant C; abnormal colorectal cancer screening)

Regardless of the understanding of the screening results, all participants reported receiving or scheduling follow‐up care. For example, Participant B (abnormal colorectal cancer screening) stated *“they* [healthcare providers] *checked further, and everything's fine”* and Participant E (abnormal cervical cancer screening) stated, “I had the colposcopy… like, a week after they called me with those res—with the positive results from their office.”

#### 2.2.2 Theme 2: Satisfaction With Self‐Sampling for Cancer Screening

Participants reported an overall positive experience with using the self‐sampling tests for cancer screening. Several participants mentioned that they liked that they could do the tests privately:

I love it, because … it's easier. It's more private. It's more convenient, because you don't have to make an appointment … (Participant C; abnormal colorectal cancer screening)

Two participants reflected on the ease of using the self‐sampling tests compared to potential difficulties (e.g., reluctance, discomfort) in completing in‐clinic cancer screening:

I have a very difficult time going to the OB‐GYN. So I just don't. And because I was in the group that got the test [in the parent study], I was very excited. Because I wouldn't have to go. This was like get my doctor to stop bugging me about going! (Participant B; abnormal colorectal cancer screening)

People, you know, don't like going to the doctor's, because it is a little humiliating, and this will somewhat eliminate that. (Participant C; abnormal colorectal cancer screening)

Participants reflected that these benefits could encourage future cancer screening, both for themselves (“I would definitely be more open to doing more home tests for screenings if there were such tests available,” Participant E; abnormal cervical cancer screening) and for other people (“There are a lot of people that can't get to health care services… this would be very helpful to them,” Participant B; abnormal colorectal cancer screening).

However, some participants identified potential barriers or drawbacks for this testing modality. For example, Participant D (abnormal colorectal cancer screening) noted that patients may not *“follow up* [on their self‐sampled cancer screening] *with a doctor, even though they may be having some other problems.”* In addition, Participant C (abnormal colorectal cancer screening) expressed concerns about mail delays: “If there was going to be a barrier, you know, unfortunately, once in a great, great while that things get lost in the mail.”

### 2.3 Mixed Methods Analysis

Upon mixed methods analysis, we identified instances of convergence and divergence between quantitative and qualitative findings. First, in terms of understanding and following up on abnormal results, we noted that cancer screening knowledge scores resonated with quotes about understanding screening results (Table 2). That is, the participants (B and C; both with abnormal colorectal cancer screening) who indicated the greatest understanding of their abnormal results had high scores for screening knowledge, suggesting that baseline cancer screening knowledge supported understanding of abnormal screening results. In contrast, although we expected to see that higher health literacy would be associated with greater understanding of results, no clear pattern was evident. Because all participants received appropriate follow‐up care, relationships between the quantitative and qualitative data related to this outcome could not be detected.

**TABLE 2:** Joint display of quantitative and qualitative data related to the theme of understanding and follow‐up on abnormal results from self‐sampled cancer screening tests.

Domain	Participant A	Participant B	Participant C	Participant D	Participant E
Cancer screening test results					
Colorectal	*Abnormal*	*Abnormal*	*Abnormal*	*Abnormal*	Normal
Cervical	Normal	Normal	Normal	Normal	*Abnormal*
Cancer screening knowledge^a^	2	3	3	2	3
Health literacy^b^	20	17	17	16	19
Illustrative quotes					
Understanding of abnormal screening results	That something was wrong because, you know, I'm‐‐ I'm … So that‐‐ I‐I‐just I make, I make appointments.	Well, they told me that that was less than normal progression of this, and I have an uncle who died of colon cancer. And I know that when you catch that early that most of the time you can live through it.	That [I] did not have the necessary markers for cervical cancer and had blood in my stool, but that could be from hemorrhoids. It could be, you know, lot of other things.	And I really didn't express concerns at all, um, because I just was spotting a little bit. I didn't know that that was a‐‐ a huge deal after menopause. But apparently it is.	They just said the results were [abnormal]. And I took that at face value and didn't ask any further questions, which I probably should have.
Receipt of appropriate follow‐up care	Um, I … needed to see a, a, a gastroenterologist.	I had one polyp and they removed it, and it was benign. They checked further, and everything's fine.	It was like, OK, Doc, you got a test. Be happy seeing you in a couple of years.	Everything came back clear, so [the healthcare provider] is not overly concerned about it, either.	I had the colposcopy… like, a week after they called me with those res—with the positive results from their office.

^a^ Potential range of scores = 0–4.

^b^ Potential range of scores = 4–20.

Second, we examined quantitative and qualitative data on satisfaction with self‐sampling for cancer screening. Because quantitative data on satisfaction with screening were uniformly high (i.e., scores of 4.0 on a range from 0 to 4), we could not evaluate these data in reference to the qualitative outcomes. However, we identified strong convergence between preference for the next cancer screening test and positive evaluation of self‐sampling, particularly for cervical cancer (Table 3). Three participants (B and C, both with abnormal colorectal cancer screening, and E, with abnormal cervical cancer screening) indicated that their preference for their next cervical cancer screening would be the at‐home test only, and in their illustrative quotes, all three specifically contrasted the self‐sampled test with in‐clinic screening (e.g., Participant B, abnormal colorectal cancer screening: “I have a very difficult time going to the OB‐GYN”; Participant C, abnormal colorectal cancer screening: “…nothing like going to the doctor's, and just… being spread‐eagled”; Participant E, abnormal cervical cancer screening: “it wasn't physically as uncomfortable—as an in‐office test”). The remaining participants were also willing to do an at‐home test for their next cervical cancer screening, but their reasons were more related to saving time (e.g., Participant A, abnormal colorectal cancer screening: “I'm a little bit out in the country and I travel by bus”; Participant D, abnormal colorectal cancer screening: “…we have busy lives”). Thus, preferences and positive evaluations for using the self‐sampling tests appear to be linked by reducing discomfort associated with cervical cancer screening, as well as reducing the time burden associated with cancer screening more generally.

**TABLE 3:** Joint display of quantitative and qualitative data related to the theme of satisfaction with the process of self‐sampling for cancer screening tests.

Domain	Participant A	Participant B	Participant C	Participant D	Participant E
Cancer screening test results					
Colorectal	*Abnormal*	*Abnormal*	*Abnormal*	*Abnormal*	Normal
Cervical	Normal	Normal	Normal	Normal	*Abnormal*
Preference for next cancer screening					
Cervical	Both at‐home and in‐person tests	At‐home test	At‐home test	No preference	At‐home test
Colorectal	Don't know	Both at‐home and in‐person tests	At‐home test	At‐home test	At‐home test
Illustrative quotes					
Positive evaluation of self‐sampling for cancer screening	Since I'm a little bit out in the country and I travel by bus, I like doing it, the home test.	I have a very difficult time going to the OB‐GYN. So I just don't. And because I was in the group that got the test [in the parent study], I was very excited. Because I wouldn't have to go. This was like get my doctor to stop bugging me about going! It allowed me to go further and have a peace of mind that everything's OK.	[It] made it more comfortable, more easy, less embarrassing, because nothing like going to the doctor's, and just … being spread‐eagled. I love it, because … it's easier. It's more private. It's more convenient, because you don't have to make an appointment, and it's accomplishing the same thing.	I think it's a great idea. I think there are a lot of women out there who would‐‐ would definitely be more open to using this and‐‐ as an initial screening… because we're busy, we have busy lives.	I was uncomfortable at the thought of all of it at first. … But having done it once, it would be a lot less uncomfortable. And, um, I‐‐ I actually like it. I mean, it‐‐ ‘cause it‐‐ it‐‐ it wasn't physically as uncomfortable‐‐ as an in‐office test.
Potential drawbacks of self‐sampling for cancer screening			If there was going to be a barrier, you know, unfortunately, once in a great, great while that things get lost in the mail.	With the cervical cancer one, that was test‐‐ testing only for HPV. And so some women may get that result back and think that they're fine, gynecologically speaking.	[Patients may not] follow up [on their self‐sampled cancer screening] with a doctor, even though they may be having some other problems.

## 3 Discussion

Our mixed methods analyses revealed that participants differed in their understanding of their abnormal results, which was related to their cancer screening knowledge; however, regardless of their level of understanding, all participants received appropriate healthcare to follow up on their abnormal screening result. In addition, satisfaction with self‐sampling for cancer screening was uniformly high, and these positive experiences led many participants to prefer a self‐sampled test for their next cancer screening.

Based on previous studies [23], we expected that understanding/knowledge would be a necessary prerequisite to pursuing and receiving diagnostic follow‐up after an abnormal screening result. However, we found that, while understanding of abnormal cancer screening results differed, 100% of participants received follow‐up care after their abnormal screening. That is, participants participated in follow‐up care, regardless of their understanding of the abnormal screening results. Notably, receipt of follow‐up care in this study exceeded the typical levels, which is ~47% for colorectal cancer screening (with FIT) [2, 3] and ~80% for cervical cancer screening (with clinician‐collected or self‐sampled hrHPV tests) [6]. This higher‐than‐expected receipt of follow‐up care is partly attributable to the fact that cancer screening was undertaken in the context of a randomized clinical trial with buy‐in from FQHC partners and devoted funding for study activities. As part of this study, a physician at the FQHC spoke with each participant about the need for follow‐up diagnostic procedures; thus, even though participants may not have been able to fully articulate the mechanism of carcinogenesis, all of them were apparently convinced of the need for early detection and diagnostic resolution. Additional research is needed to identify sustainable strategies to ensure receipt of follow‐up diagnostic services for abnormal screenings detected with self‐sampling tests, particularly among patients with barriers to routine care. Educational interventions are an attractive strategy, but although increasing knowledge about cancer screening can promote understanding of abnormal results, for participants in this study, knowledge and understanding were not necessarily related to receipt of follow‐up care.

Among general patient populations, satisfaction with self‐sampling is very high for colorectal cancer screening [24] and for cervical cancer screening [7, 8]. However, the experience of receiving an abnormal cancer screening result can be quite aversive [25, 26], which can impact satisfaction with screening and even reduce the likelihood of repeat screening in the future [26, 27]. Given that our study focused on a subgroup of patients with abnormal cancer screening results, all of whom ultimately received follow‐up care and were not diagnosed with cancer, we expected that this experience would negatively impact satisfaction. Despite this possibility, satisfaction with the self‐sampling cancer screening process was uniformly high, and most participants preferred to use a self‐sampling tool again for their next cancer screening. Notably, although only 60% of participants reported that they would prefer an at‐home test for their next screening, the remaining participants indicated no preference or a preference for both an at‐home and in‐person test; that is, none of the participants preferred to undergo screening in the future using only an in‐person test. The qualitative data highlighted the positive experiences participants had with self‐sampling, particularly for cervical cancer screening. Given that widespread cancer screening will always produce some number of false‐positive results [28], our findings are encouraging in that these abnormal results from self‐sampled tests (which were ultimately not diagnosed as cancer) did not unduly prejudice participants against the entire cancer screening process.

### 3.1 Implications

Improving quality and access for “near home” cancer services is a public health priority, including increasing use of self‐sampling tests for cancer screening [29]. Self‐sampling tests for colorectal cancer screening have been included in USPSTF guidelines for many years [4], and self‐sampling tests for cervical cancer screening may be incorporated into guidelines in the future [30]. These tests may be particularly attractive to low‐income, underinsured patients who are less likely to be up‐to‐date with screening [5]. However, screening initiation is only one step in the screening continuum, and failure to follow up on abnormal screening results can lead to avoidable morbidity and mortality [31]. Some healthcare providers are reluctant to embrace self‐sampling for cancer screening because of anticipated challenges with delivering follow‐up care to patients who use this testing modality [8, 9]. In this study, we sought to evaluate experiences with self‐sampling and accessing follow‐up care among FQHC patients with abnormal screening results. We found that participants were able to access follow‐up diagnostic care, and that they remained highly satisfied with the self‐sampling cancer screening process. These findings provide additional support for the potential impact of expanding access to self‐sampling cancer screening tests, as long as they are accompanied by strategies to connect patients with diagnostic follow‐up care in the event of abnormal results. Specifically, our findings demonstrate that follow‐up care is more closely related to support from the clinic and healthcare team than it is to knowledge about the screening process. In the long term, these efforts can reduce avoidable cancer mortality, both for the overall population and for patients adversely affected by health disparities.

### 3.2 Limitations and Strengths

Our sample size was limited by the small number of participants who received abnormal screening results in the parent study. To mitigate this limitation, we integrated quantitative and qualitative findings to support a more robust, in‐depth investigation into participants' experiences. A related limitation is the demographic homogeneity of the sample; future research should explore these findings in larger, more diverse samples. Further, social desirability bias may have impacted the results. Specifically, participants may have responded to survey and interview items with a more positive perspective than their true attitudes [32] because they were aware that our study team was interested in the potential impact of self‐sampling tests. In addition, as noted, our study was conducted in the context of a highly controlled research study, resulting in procedures that may not be sustainable in real‐world clinical settings. Future studies should leverage larger samples with more pragmatic procedures to replicate our study findings.

In terms of study strengths, we used a mixed methods approach to contextualize and enrich our scientific understanding of patient experiences after receiving abnormal results on a self‐sampled cancer screening test. Further, our study focused on an understudied population, and findings can be used to improve care and reduce healthcare disparities impacting low‐income, underinsured patients.

## 4 Conclusions

We conducted a mixed methods analysis of FQHC patients' experiences with self‐sampling for cancer screening after receiving abnormal results. Despite differing levels of understanding of the abnormal results, all participants were able to access follow‐up diagnostic care. Further, participants had very high satisfaction with the entire self‐sampling cancer screening process. These findings support the potential impact of public health priorities focused on expanding access to self‐sampling tests, especially for increasing uptake and follow‐up care, and reducing avoidable cancer mortality.

## Author Contributions

**Jennifer L. Moss:** conceptualization (lead), data curation (equal), formal analysis (equal), funding acquisition (lead), investigation (lead), methodology (lead), project administration (equal), resources (equal), software (equal), supervision (equal), visualization (equal), writing – original draft (lead), writing – review and editing (lead). **Veronica E. Bernacchi:** data curation (equal), formal analysis (equal), investigation (equal), software (equal), validation (equal), writing – original draft (equal), writing – review and editing (equal). **Juliette Entenman:** formal analysis (equal), investigation (equal), visualization (equal), writing – original draft (supporting). **Heather Costigan:** data curation (equal), formal analysis (equal), investigation (equal), validation (equal), writing – original draft (supporting). **Heather L. Stuckey:** conceptualization (equal), formal analysis (equal), investigation (equal), project administration (equal), supervision (equal), writing – original draft (equal), writing – review and editing (equal). **Mack T. Ruffin:** conceptualization (equal), funding acquisition (equal), resources (equal), writing – original draft (equal), writing – review and editing (equal).

## Conflicts of Interest

The authors declare no conflicts of interest.

## Supporting information


**Table S1:** In‐depth interview guide examining experiences with self‐sampling for cancer screening, among participants who received abnormal results on a screening test, as part of a parent randomized controlled trial.

**Table S2:** Descriptive statistics for sociodemographic characteristics of participants in the parent study who were randomized to receive self‐sampling for cancer screening and of participants who received abnormal screening results and participated in the current follow‐up study.

## Data Availability

The data that support the findings of this study are available on request from the corresponding author. The data are not publicly available due to privacy or ethical restrictions.
