# Supplementary material for: Follow‐Up After Receiving Abnormal Results From Self‐Sampled Colorectal and Cervical Cancer Screening Tests Among Underserved Patients
Source: Cancer Med. 2025 Sep 27;14(19):e71283. doi: 10.1002/cam4.71283 (PMC12475505; doi:10.1002/cam4.71283)
Supplement: Supplementary file 1 — Table S1: In‐depth interview guide examining experiences with self‐sampling for cancer screening, among participants who received abnormal results on a screening test, as part of a parent randomized controlled trial. [file CAM4-14-e71283-s001.docx]

**Supplemental Table S1.** In-depth interview guide examining experiences with self-sampling for cancer screening, among participants who received abnormal results on a screening test, as part of a parent randomized controlled trial.

| **Main question** | **Potential probes** |
| --- | --- |
| How did the clinic share the results of the test with you? | Did they contact you by phone? Mail? Electronic health record portal? In-person at a healthcare visit? Do you remember when you heard from the clinic? How long did you wait between sending in the test results and finding out the results? Who talked to you about your results? |
| What was your understanding of the test results? | What did the clinic say about your results? Did they give you an opportunity to think and ask questions about your results? What do you think the test results mean for your health? |
| What did the clinic recommend you do to follow-up on this test? | Did they suggest that you redo the test? Do additional tests? Did they suggest you visit the clinic to talk to your provider? |
| How did you decide what to do to follow-up on this test? | Did you talk to anyone in your family about the test results? Any friends or neighbors? Did you do any additional research, e.g., online? |
| Since getting the results of this test, what additional care have you received at this clinic or any other clinic? | Did you get a follow-up test to evaluate your cancer risk? Did you go to a clinic to get a check-up, or for any other care? Did you receive any diagnosis after getting the test results? What sort of barriers have you faced in getting follow-up for these test results? |
| Now I’d like you to think about doing the home test to check for cancer, and everything that came after. How do you think the home test impacted this process? | How did home testing help you get tested for cancer, and get follow-up? How did home testing create barriers in getting tested for cancer, and getting follow-up? |
| How has home testing changed the way you think about getting tested for cancer? | Do you plan to continue getting tested for cancer in the future? There are some tests for cancer that can only happen at the provider’s office, like a mammogram. How likely are you to get checked for cancer at the provider’s office? |
| Overall, what do you think about the home testing process? | Would you do the test at home again, if available and recommended by your provider? Would you recommend home testing to other people in your life? |
