# Supplementary material for: Follow‐Up After Receiving Abnormal Results From Self‐Sampled Colorectal and Cervical Cancer Screening Tests Among Underserved Patients
Source: Cancer Med. 2025 Sep 27;14(19):e71283. doi: 10.1002/cam4.71283 (PMC12475505; doi:10.1002/cam4.71283)
Supplement: Supplementary file 2 — Table S2: Descriptive statistics for sociodemographic characteristics of participants in the parent study who were randomized to receive self‐sampling for cancer screening and of participants who received abnormal screening results and participated in the current follow‐up study. [file CAM4-14-e71283-s002.docx]

**Supplementary Table S2.** Descriptive statistics for sociodemographic characteristics of participants in the parent study who were randomized to receive self-sampling for cancer screening and of participants who received abnormal screening results and participated in the current follow-up study.

|  | Participants in parent study (self-sampling arm; *n*=24) | | Participants in current study (*n*=5) | |
| --- | --- | --- | --- | --- |
|  | ***n*** | **%** | ***n*** | **%** |
| Race/ethnicity |  |  |  |  |
| Non-Hispanic white | 20 | 83% | 4 | 80% |
| Other | 4 | 17% | 1 | 20% |
| Annual household income |  |  |  |  |
| <$50,000 | 17 | 71% | 4 | 80% |
| $50,000+ | 7 | 29% | 1 | 20% |
| Educational attainment |  |  |  |  |
| High school degree or less | 8 | 33% | 1 | 20% |
| Greater than high school degree | 16 | 67% | 4 | 80% |
| Marital status |  |  |  |  |
| Married/living as married | 14 | 58% | 2 | 40% |
| Other | 10 | 42% | 3 | 60% |
| Last-year preventive health check-up |  |  |  |  |
| No | 7 | 29% | 1 | 20% |
| Yes | 17 | 71% | 4 | 80% |
| Health insurance type |  |  |  |  |
| Private | 6 | 25% | 1 | 20% |
| Other | 18 | 75% | 4 | 80% |

*Note.* All *p*>.05 for Fisher’s exact tests comparing participants from the parent study who did not versus did participate in the current study.
